# Supplementary material for: Ranolazine Attenuates Brain Inflammation in a Rat Model of Type 2 Diabetes
Source: Int J Mol Sci. 2022 Dec 18;23(24):16160. doi: 10.3390/ijms232416160 (PMC9782607; doi:10.3390/ijms232416160)
Supplement: Supplementary file 1 [file ijms-23-16160-s001.zip › ijms-2031357-supplementary.pdf]

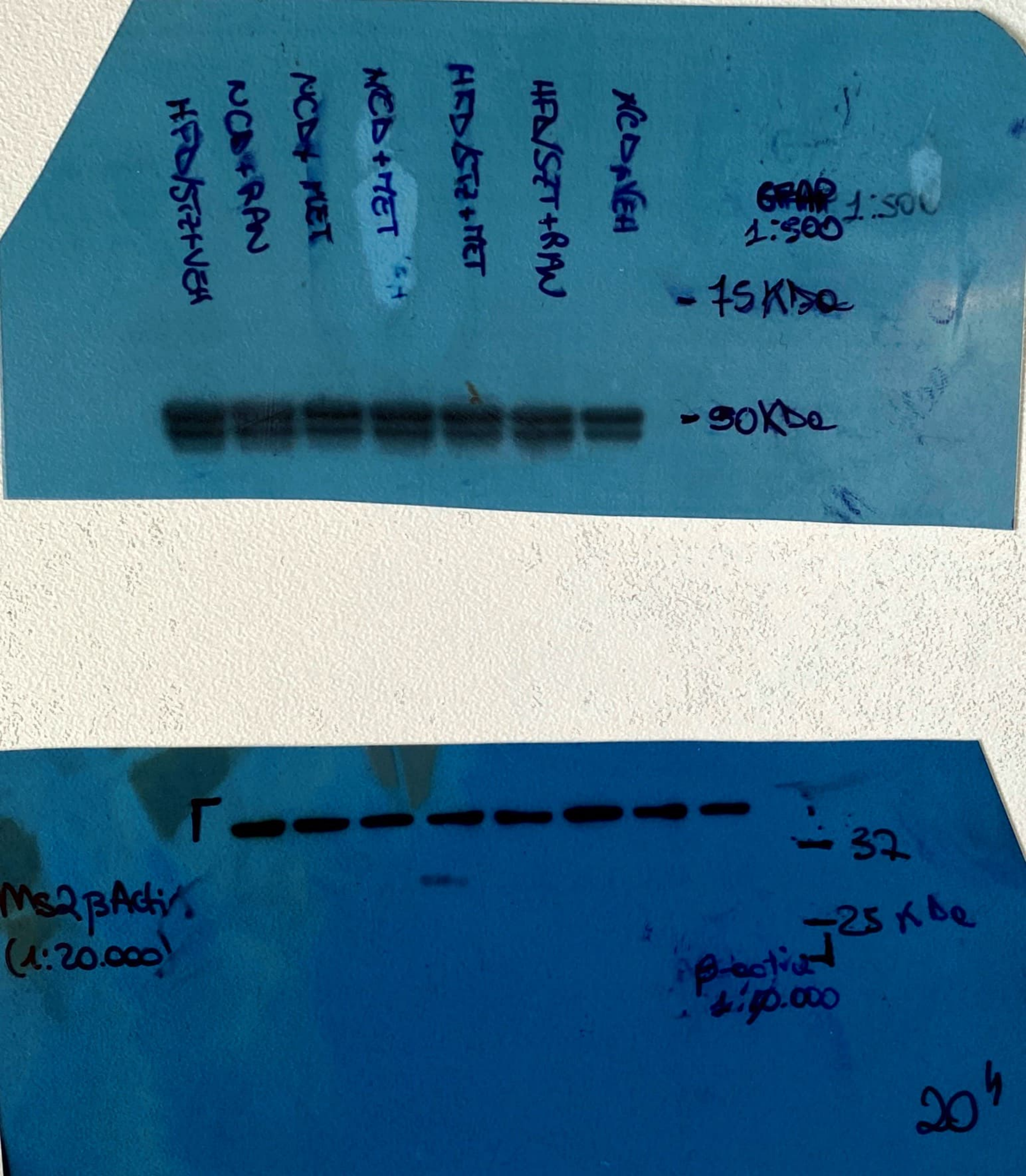
HFD/STZ+VEH
NCD+RAW
NCD+MET
NCD+MET
HFD/STZ+MET
HFD/STZ+RAW
NCD+VEH
GFAP 1:500
1:500
75 KDa
50 KDa
37
25 KDa
Ms2βActin (1:20.000)
β-actin 1:20.000
20''

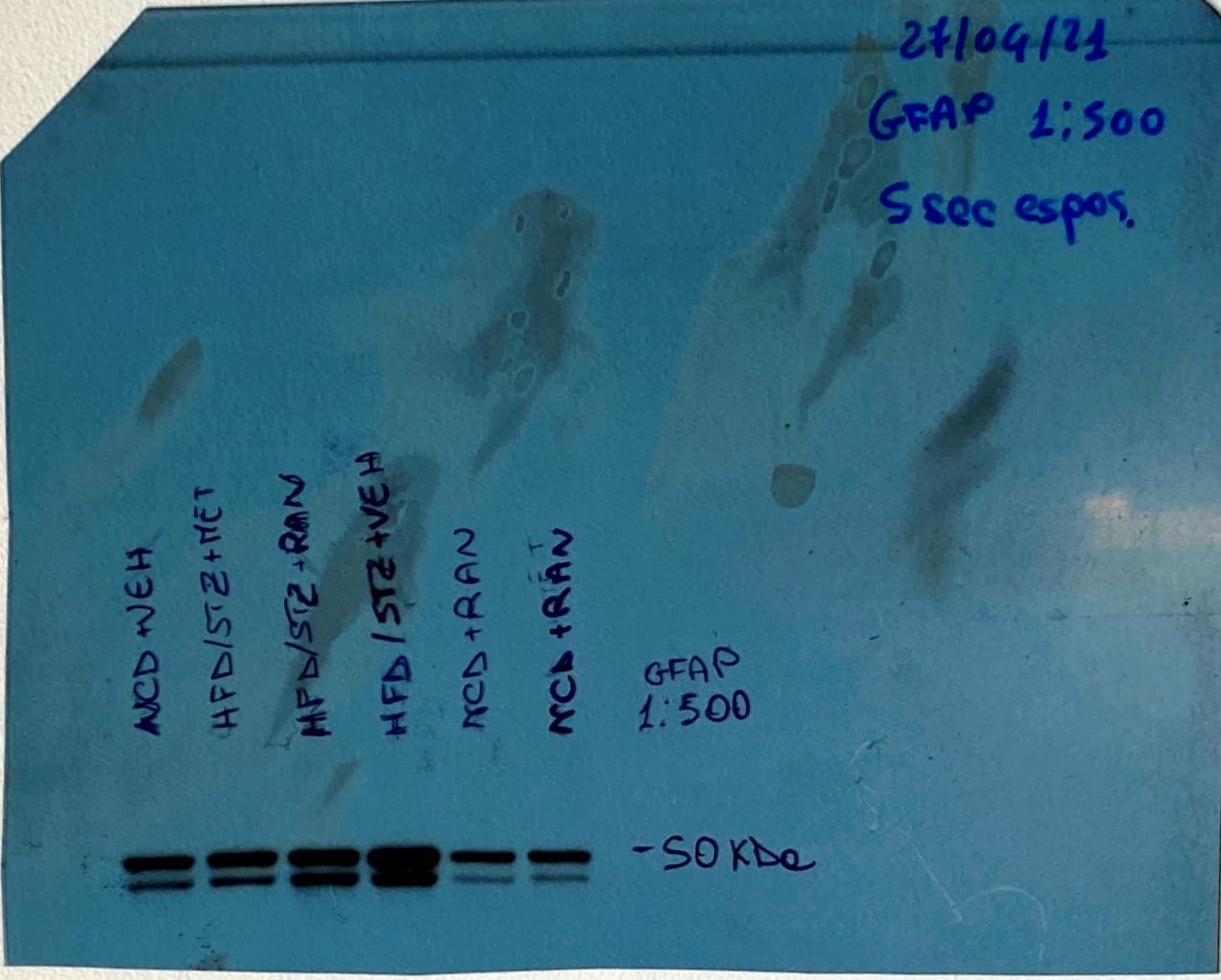
27/04/21
GFAP 1:500
5 sec espos.
NCD+VEH
HFD/STZ+MET
HFD/STZ+RAN
HFD/STZ+VEH
NCD+RAN
NCD+RAN
GFAP
1:500
-50 KDa

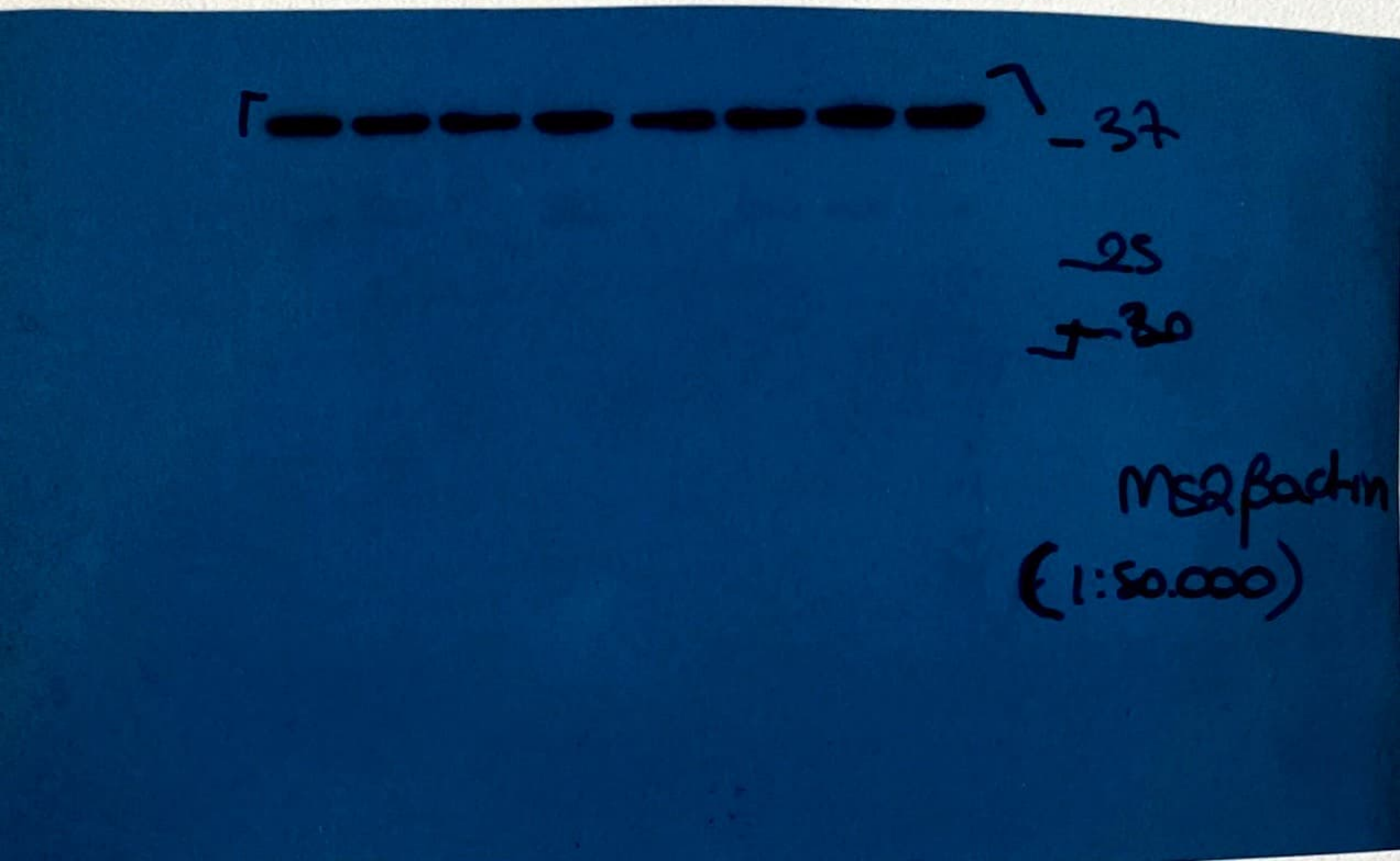
37
25
30
Msα βactin
(1:50.000)

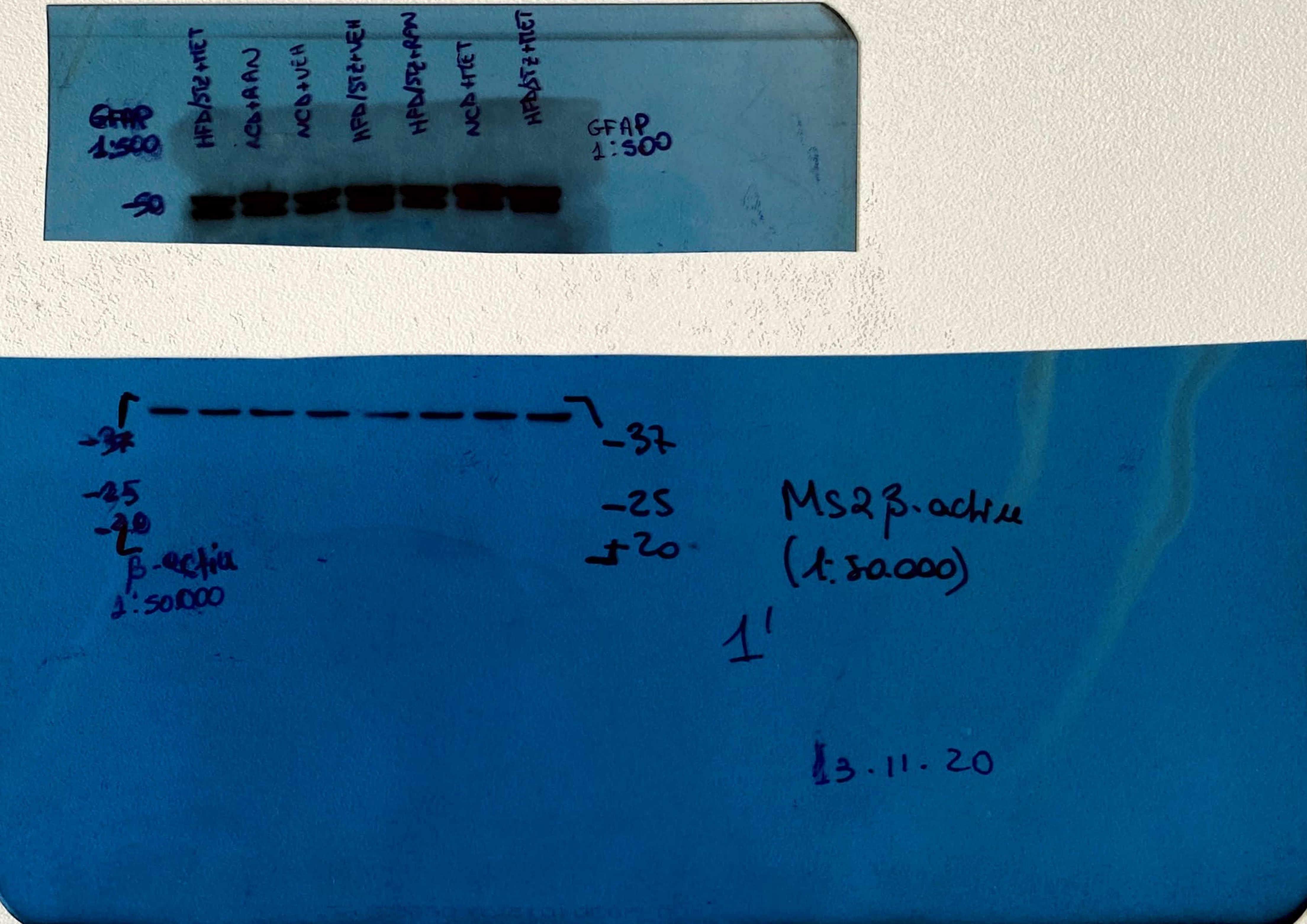
GFAP 1:500
HFD/STZ+MET
NCD+RAN
NCD+VEH
HFD/STZ+VEH
HFD/STZ+RAN
NCD+MET
HFD/STZ+MET
GFAP 1:500
-50
-37
-25
-20
β-actin 1:50.000
-37
-25
-20
Ms2β.actin (1:50.000)
1'
13.11.20
